# Stabilizing Li-metal host anode with LiF-rich solid electrolyte interphase

**DOI:** 10.1186/s40580-021-00269-4

**Published:** 2021-06-14

**Authors:** Jaewoo Lee, Min-Sik Park, Jung Ho Kim

**Affiliations:** 1 Institute for Superconducting and Electronic Materials (ISEM), Australian Institute of Innovative Materials (AIIM), University of Wollongong, Innovation Campus, Squires Way, North Wollongong, NSW 2500 Australia; 2 Department of Advanced Materials Engineering for Information and Electronics, Integrated Education Program for Frontier Materials (BK21 Four), Kyung Hee University, 1732 Deogyeong-daero, Giheung-gu, Yongin, 17104 Republic of Korea

**Keywords:** Li-metal anode, Zeolitic imidazolate framework, Cobalt catalyst, Lithium fluoride, Solid-electrolyte interphase

## Abstract

The development of lithium (Li)-metal anode is high priority research to initiate next-generation Li batteries. Applying Li-metal in practical applications as anode still has many hurdles to clear away, such as low Coulombic efficiency and capacity degradation by the continuous formation of dead Li. We demonstrate that cobalt (Co) nanoparticle incorporation in a porous carbon host anode can play a critical role in the formation of a thick lithium fluoride dominated solid-electrolyte interphase in ether-based electrolyte. As a result, the host anode containing Co nanoparticles shows excellent electrochemical performance with high Li-metal reversible capacity and even stable long-term cyclability with no dead Li formation.

## Introduction

For the past few decades, lithium (Li) secondary batteries have played a crucial role in facilitating more rapid growth of portable electrical devices and the electric vehicle market, because their energy density and operating voltage are considerably higher than those of previous secondary batteries [1–4]. In recent years, however, such batteries cannot meet the increasing demand for energy storage devices due to the difficulties in further progress with the current transition metal oxide/graphite system [5]. To resolve this issue, exploration of new advanced materials is required to achieve a major breakthrough, which will require entering the stage of next-generation Li batteries [6].

From the point of view of anode materials, Li-metal is regarded as the most promising alternative to replace the current graphite or graphite/silicon composite anodes due to its substantially high theoretical capacity (3860 mAh g^−1^) [7, 8], although applying Li-metal in practical applications as an anode has still many hurdles to clear away. For instance, repeated Li plating/stripping during Li-metal anode cell operation forms dendritic Li and irreversible Li (called dead Li), leading to internal short-circuiting and capacity fading [9, 10]. To resolve such problems, for one example, strategically designed host structures for stable Li-metal storage need to be suitably introduced with surface chemistry that has a strong affinity with Li [11–18].

Based on above aspects, we have reported that the physical properties of nanoporous zeolitic imidazolate framework (ZIF)-derived carbons can be controlled by different ratios of zinc (Zn) to cobalt (Co)-ion metallic precursors [19–21]. This approach was designed to achieve tailored ZIF-derived carbons with different pore volumes, pore sizes, surface areas, and even degrees of graphitization. Owing to their large pore volumes and adjustable physical properties, it is suggested that the electrochemical performances of these ZIF-derived carbons as Li-metal storage hosts can be maximized by controlling these properties. Moreover, as reported [22], by controlling the synthesis of bimetallic ZIF-derived carbon, Co nanoparticle embedded highly mesoporous carbon is proposed as a new innovative Li-metal host anode. This material shows enhanced Li affinity and superior electrochemical performance. According to the density functional theory calculations, Co-assisted nitrogen (N)-doped graphite structures can facilitate delocalization of transferred electrons from Li atoms near Co–N. This indicates that the strong binding and interaction between carbon and Li can be affected by the delocalized electrons over a wide range of area on the graphite surface, although it still remains elusive as to how Co nanoparticles affect the interphase between ether-based electrolyte and the ZIF-derived carbon electrode. Thus, in-depth analysis to determine the solid basis for the phenomena involving different Li-metal growth behavior is required under different surface environments.

Herein, by a comparative study on two ZIF-derived carbon candidates, which are distinctively different in the presence and absence of Co nanoparticles, we propose a significant clue that evenly distributed Co nanoparticles can play an immediate role in forming a thick and rich lithium fluoride (LiF) dominated solid-electrolyte interphase (SEI) on the surface of the electrode. Moreover, once the LiF-SEI is firmly established on the electrode surface, induced by the synergetic interaction between the Co nanoparticle-containing medium and the ether-based electrolyte, the anode shows excellent electrochemical performance with high Li-metal reversible capacity and even stable long-term cyclability with no dead Li formation.

## Experimental

### Materials preparations

**Fig. 1:**
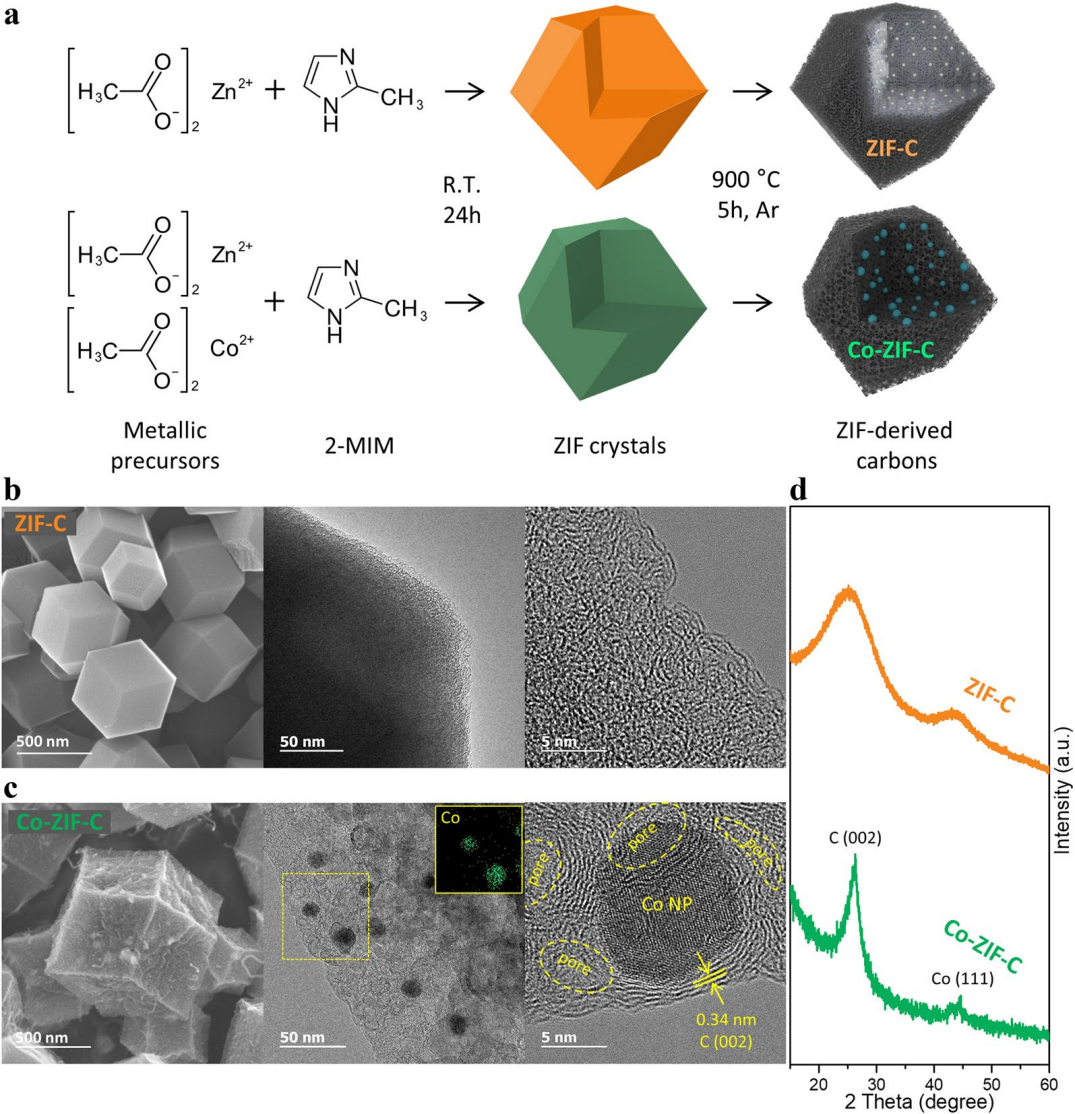
**a** Schematic illustration of the synthesis of ZIF-derived carbons, FESEM and TEM images of (**b**) ZIF-C and **c** Co-ZIF-C; **d** XRD patterns of ZIF-C and Co-ZIF-C

To synthesize ZIF-derived carbon, specific atomic ratios (Zn:Co = 1:0 for ZIF-C and 1:2 for Co-ZIF-C) of the zinc acetate dihydrate (2-*x* g, Sigma-Aldrich) and/or cobalt acetate tetrahydrate (*x* g, Sigma-Aldrich) were dissolved in water and stirred for 10 min in a 2-methylimidazole (10 g, Sigma-Aldrich) solution. Each solution was maintained at room temperature for 24 h. The precipitates were then collected by filtering and drying in an oven at 80 ℃ for 24 h. The obtained powder was heat-treated at 1000 ℃ for 6 h under argon gas with a ramping rate of 5 ℃ min^−1^. Afterwards, the two powders were kept in 1 M hydrogen chloride (HCl) solution (100 ml) for 24 h each, and then washed several times in a large amount of water. Subsequently, the ZIF-derived carbon was finally obtained after drying (Fig. 1a) [22].

### Structural characterizations

Microscopic observations were performed using a field emission scanning electron microscope (FESEM, JSM-700F) and a transmission electron microscope (TEM, JEM-2100F and ARM-200F). X-ray powder diffraction (XRD) patterns were analyzed using an X-ray diffractometer (MMA). X-ray photoelectron spectroscopy (XPS) analysis was performed using a surface analysis system (K-Alpha X-ray XPS System).

### Electrochemical measurements

The ZIF-derived carbons electrodes were manufactured by coating a mixed active material/Super-P/polyvinylidene fluoride mixture in N-methyl-2-pyrrolidone solution with a weight ratio of 8:1:1 onto Cu foil. Coin cells (CR2032) were assembled with Li-metal foil, a polyethylene membrane, and 1 M lithium bis(trifluoromethanesulfonyl)imide (LiTFSI) in mixed dimethyl ether (DME) and 1,3-dioxolane (DOL, 50:50 in vol%), including 1 wt% lithium nitrate (LiNO_3_) as counter electrode, separator, and electrolyte, respectively. Full-cells were also assembled with LiFePO_4_ (LFP) cathode fabricated with a weight ratio of 90:5:5 (LFP: Super-P carbon black: PVDF binder) with an areal capacity of 0.6 mAh cm^−2^ instead of Li-metal foil to operate in the voltage range of 2.5 to 4.0 V vs. Li/Li^+^ .

## Results and discussion

### Characterizations of ZIF-derived carbons

To determine the possibility of ZIF-derived carbons as stable Li-metal host anodes, two distinctive types of ZIF-derived carbons were introduced with account of their high pore volume properties among all the other types of ZIF-derived carbons [23]. Figure 1b shows images of the morphology of ZIF-C synthesized by using only zinc acetate dihydrate as a metallic precursor. It can be seen to have a three-dimensional polyhedral architecture consisting mainly of amorphous carbon and micropores (< 2 nm). On the contrary, Co-ZIF-C synthesized by using mixed zinc acetate dihydrate and cobalt acetate tetrahydrate formed a graphitic carbon layered structure (Fig. 1c). This can be attributed to the size effect of Co nanocatalyst [20].

After the HCl washing process, relatively large Co-metal particles with a diameter of more than 50 nm were removed, and extra pores were formed where the large Co particles were originally located. Finally, polyhedral shaped 1 μm size particles were uniformly synthesized. From the TEM analysis, it was confirmed that randomly, but evenly distributed Co nanoparticles intertwined with the graphite layers remained intact. The XRD patterns also clearly show graphitic crystalline peaks and metallic cobalt peaks for Co-ZIF-C, while ZIF-C shows broad peaks, indicating a typical amorphous carbon structure, as shown in Fig. 1d [20].

### Electrochemical measurements of ZIF-derived carbons

Electrochemical tests of both ZIF-C and Co-ZIF-C electrodes were evaluated using 1M LiTFSI in DME/DOL including LiNO_3_ as an electrolyte to maximize cell performance in the case of Li-metal anode. This is because ether-based electrolytes can endow Li metal anode with more prolonged cycling [24, 25]. This type of electrolyte can facilitate the practicality of Li metal anode and the stability of the material, especially since LiNO_3_ and the fluorine-containing salt help to improve the surface chemistry of the Li metal anode interphase [26].

**Fig. 2:**
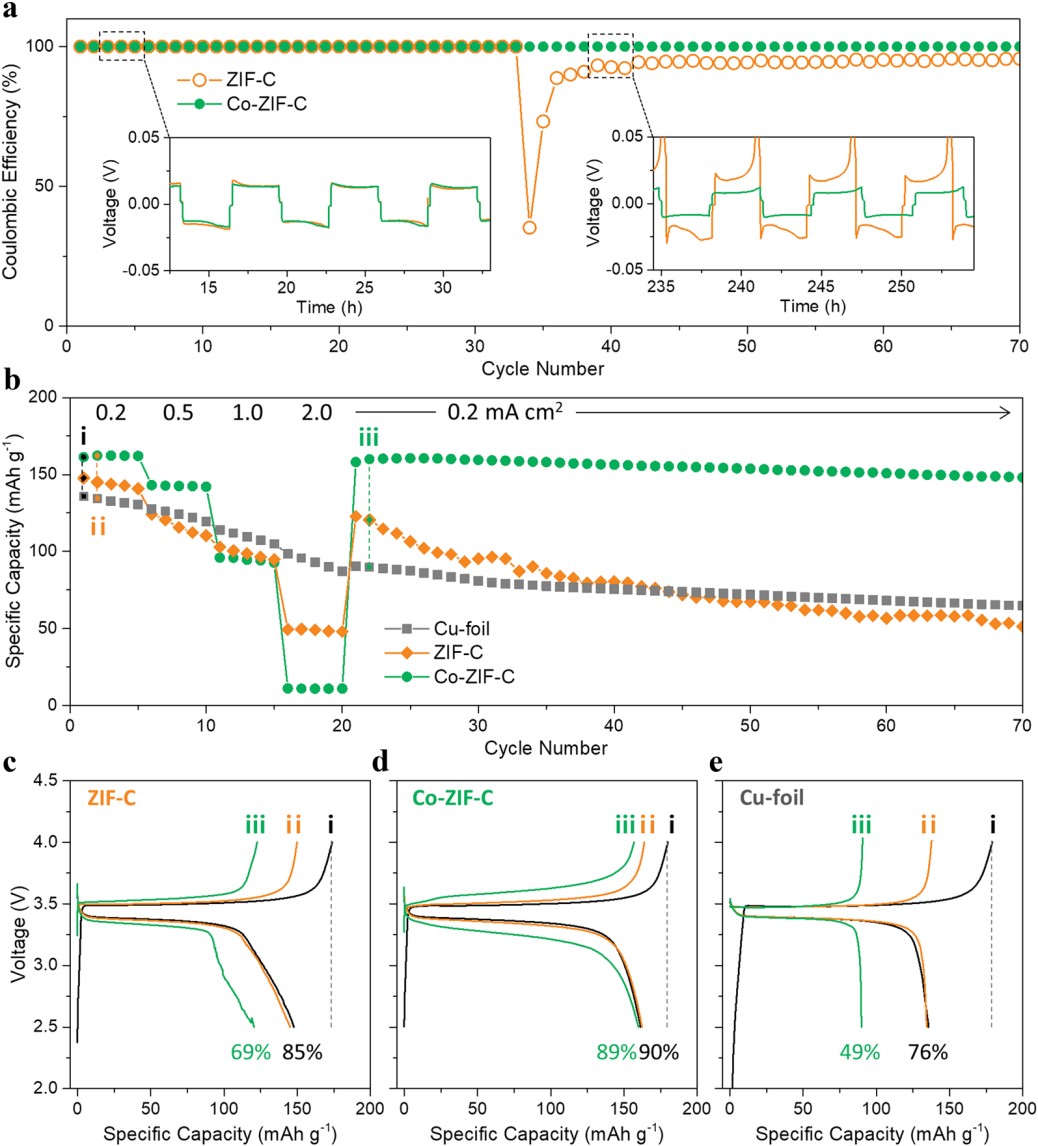
**a** Coulombic efficiencies of each cycle for the ZIF-C and Co-ZIF-C electrodes (half-cells) and their voltage hysteresis observations (in insets), **b** full-cell test results at different current densities, and the charge–discharge plots of (**c**) ZIF-C electrode, **d** Co-ZIF-C electrode, and **e** Cu-foil reference electrode

To determine the reversibility during repeated plating and stripping, half-cell tests of ZIF-C and Co-ZIF-C electrodes were evaluated under an areal capacity of 0.6 mAh cm^−2^ at a current density of 0.2 mA cm^−2^ (Fig. 2a). Co-ZIF-C showed reliable cycling over 70 cycles while maintaining 100% Coulombic efficiency, while ZIF-C experienced a sudden drop in Coulombic efficiency and consequential discharge capacity degradation over 30 cycles. In detail, the two electrodes showed stable performances with merely different overpotentials during first 30 cycles (Fig. 2a, left inset). ZIF-C, however, showed a typical failure mechanism after 200 h of measurement time (Fig. 2a, right inset), which is attributed to interference, with convoluted Li-ion pathways surrounded by inactive phase in the interphase between the electrode and the electrolyte [27]. This failure might be due to the phenomenon that ZIF-C electrode does not generate bulky Li dendrites on the electrode surface but forms an irregular SEI with the gradual growth of Li due to its relatively lower lithiophilicity. This gradual growth would result in continuous electrolyte consumption as well as an increase in the internal resistance [10]. The reversible Li source is, consequently, consumed by its conversion to an irreversible Li or Li-containing phase, which increases, in turn, the overpotential during repeated Li plating/stripping.

Meanwhile, capacity retention in the full-cell system clearly indicates the amount of irreversible Li formation because the Li source is only from the counter cathode, which is accurately fixed in the system. In this respect, the full-cell test results also present identical information to that interpreted from the half-cell test results. As shown in Fig. 2b, different current densities were applied, and the reversible capacities were finally recovered at the initial current density of 0.2 mA cm^−2^. However, at the relatively high current density of 2.0 mA cm^−2^, the ZIF-C electrode could not recover its original specific capacity recorded at 0.2 mA cm^−2^ with a significant loss of reversibility (85% to 69%, Fig. 2c). On the other hand, the Co-ZIF-C electrode maintained its reversible capacity, even at a high current density of 2.0 mA cm^−2^ (90% to 89%, Fig. 2d). The Cu-foil (empty host) electrode as reference also showed similar behavior to the ZIF-C electrode (76% to 49%, Fig. 2e). It is believed that a guided deposition of Li-metal can be facilitated in the Co-ZIF-C electrode. Namely, it is guided to mainly into the pores without dendritic growth of Li-metal on the surface at a high current density, whereas ZIF-C and Cu-foil reference electrodes underwent random and irregular growth of Li-metal on their surfaces at a high current density, and a certain portion of the surface grown Li-metal was transformed to irreversible Li. On the contrary, Co-ZIF-C electrode did not incur such an unwanted outer growth of Li-metal, even at a high current density.

### Microscopic observations of Li-plated electrodes

**Fig. 3:**
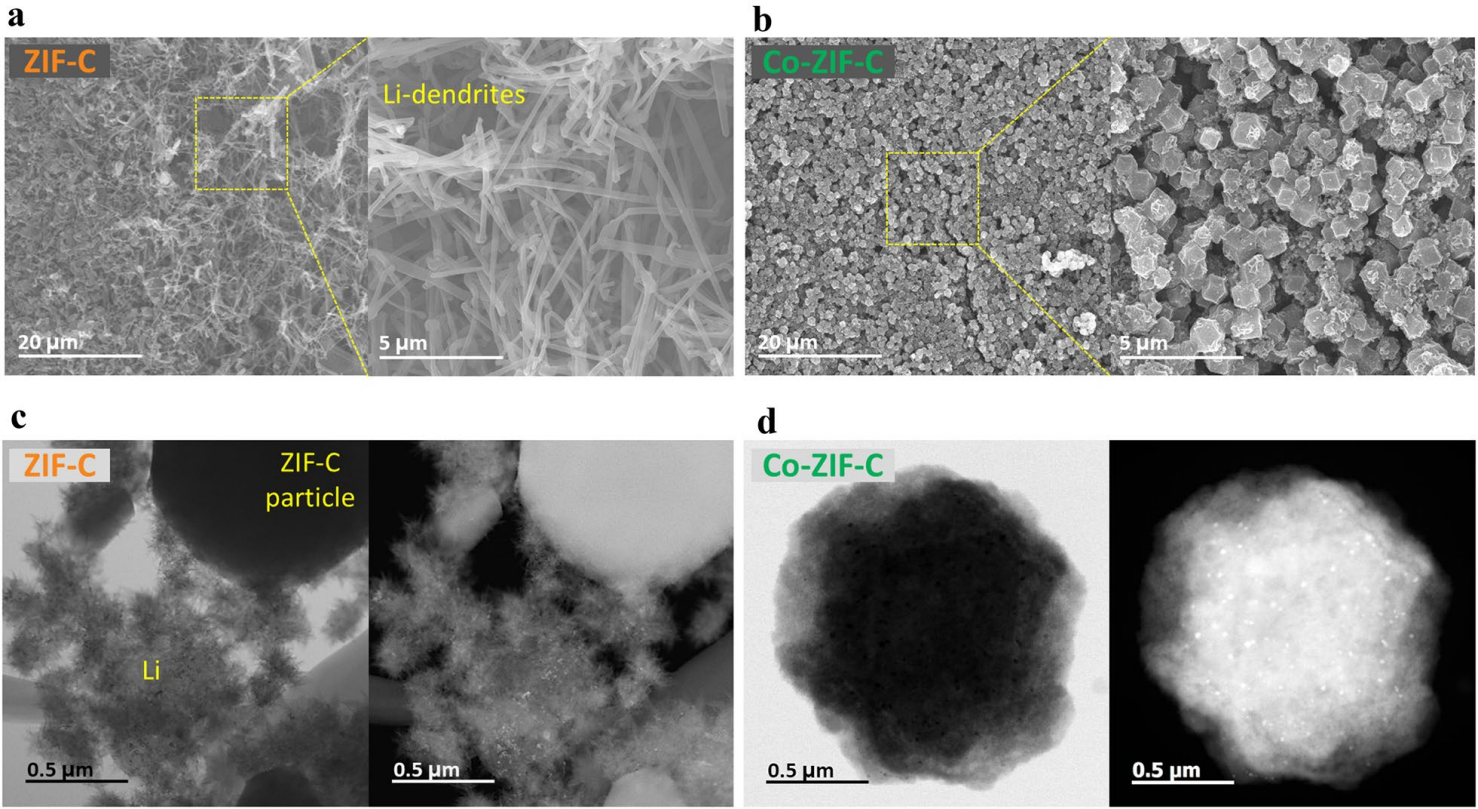
FESEM top-view observations of Li-plated (**a**) ZIF-C and (**b**) Co-ZIF-C electrodes; TEM observations of Li-plated (**c**) ZIF-C and (**d**) Co-ZIF-C particles

To understand the Li-metal forming behavior on the ZIF-derived electrodes, microscopic observations were conducted after charging to an areal capacity of 2 mAh cm^−2^ at a current density of 0.2 mA cm^−2^. Figure 3a and b shows scanning electron microscope (SEM) top-view images of ZIF-C and Co-ZIF-C electrodes, respectively. As shown in Fig. 3a, light-colored net-like Li-metal covered the surface of ZIF-C electrode. On the other hand, there was no observable Li-metal growth on the surface of the Co-ZIF-C electrode. Moreover, despite the high areal charge capacity, polyhedral-shaped Co-ZIF-C particles were still visible at all points, as for the pristine electrode. In other words, a 2 mAh cm^−2^ amount of Li-metal was stably stored in the pores of the Co-ZIF-C host. To be specific, the scanning-TEM images show large lumps of cactus-like Li-metal growth near an Li-plated ZIF-C particle (Fig. 3c), whereas dense and smooth Li metal was formed on the surface of an Li-plated Co-ZIF-C particle, as is shown, and no separated or pointed Li-metal forms could be confirmed (Fig. 3d). In consequence, these results demonstrate that the two different types of ZIF-derived carbon show distinctively different Li-metal growth behavior on their surfaces. Moreover, the Li-metal is only fully and stably settled in, both on the surface and inside the host framework of Co-ZIF-C electrode alone.

### XPS analysis of Li-plated electrodes

**Fig. 4:**
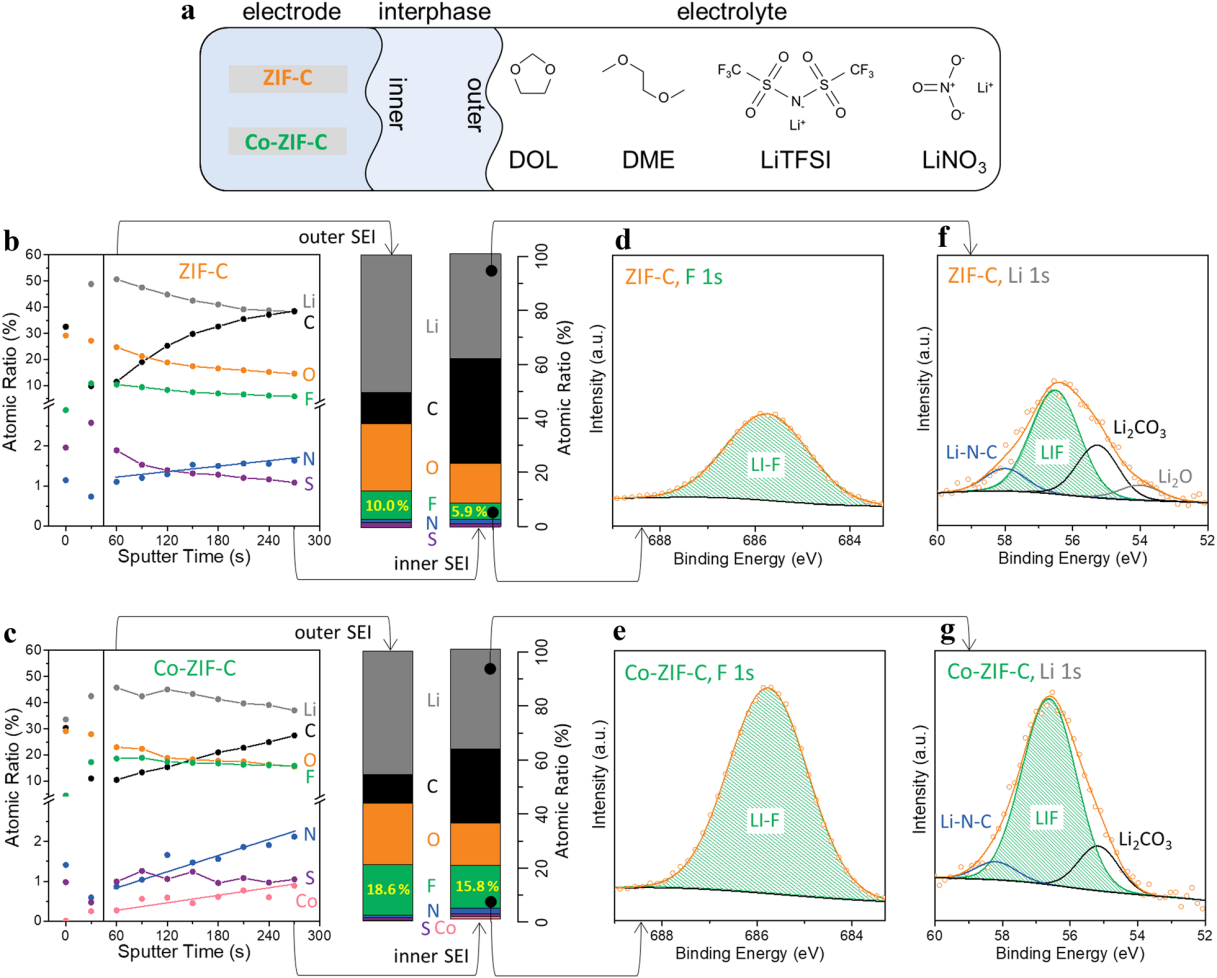
**a** Schematic illustration of the components on the ZIF-derived carbon electrodes, ether-based electrolyte, and their interphases; XPS depth profiles of Li-plated (**b**) ZIF-C and (**c**) Co-ZIF-C electrodes; F 1 s spectra of (**d**) ZIF-C and (**e**) Co-ZIF-C electrodes, and Li 1 s spectra of (**f**) ZIF-C and (**g**) Co-ZIF-C electrodes

To determine the interphase decomposition behavior under the given electrolyte condition (Fig. 4a), XPS analysis was carried out on both ZIF-C and Co-ZIF-C electrodes after charging to 2 mAh cm^−2^ at a current density of 0.2 mA cm^−2^. Figure 4b and c shows depth profiles of ZIF-C and Co-ZIF-C electrodes collected for a total of 10 times for 270 s with a 30 s interval of sputtering, respectively. The data for the outermost surface exposed to dry air (first two datapoints) were not considered in determining the trends in atomic ratio changes. When to the sputtering reached the inner phase, the ZIF-C electrode showed a gradual decrease in all other atoms in accordance with the gradual increase in carbon (C) and nitrogen (N) atoms, mainly originating from the chemical species of ZIF-C (Fig. 4b). Co-ZIF-C, however, showed an increase in C, N, and Co (Fig. 4c). Very interestingly, a 15.8% atomic ratio of fluorine (F) in the interphase of Co-ZIF-C electrode, unlike the other elements, was still retained after sputtering, down from 18.6%, whereas the F ratio for ZIF-C was nearly halved (10.0% to 5.9%). As shown in Fig. 4d and e, it was confirmed that F element in each interphase formed Li-F (685.8 eV) bonds originating from LiTFSI salt [28]. As a result of the higher atomic ratio of F maintained on Co-ZIF-C electrode, LiF accounted for the dominant portion of the interphase of Co-ZIF-C electrode (Fig. 4g), while the interphase on ZIF-C consisted of various kinds of solid Li compounds, such as Li-oxides, Li-carbonates, and N–C containing species (Fig. 4f). It is believed that the evenly distributed Co nanoparticles on Co-ZIF-C had a critical effect towards forming an LiF-rich SEI functionalized by catalytic fluorination, resulting in uniform Li deposition. Consequently, it enabled stable cycling of Li-metal anode cells [29–31].

## Conclusion

**Scheme 1:**
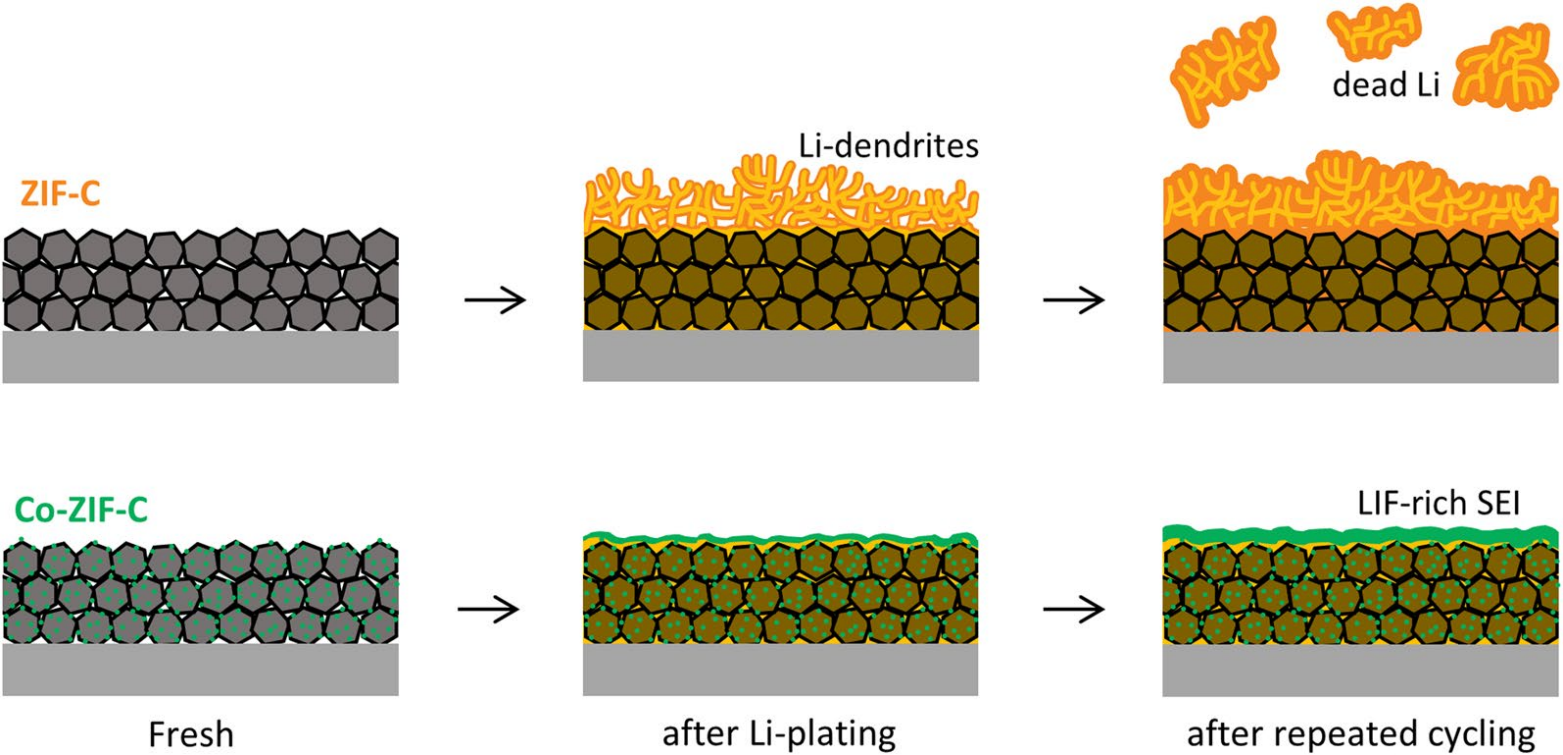
Summary of the stable cycling performance enabled by LiF-rich SEI formation on Co-ZIF-C electrode

In this study, we prepared two ZIF-derived carbons that differed in the presence and absence of Co nanoparticles, and evaluations of their structural properties and electrochemical performances with Li-plating behavior were carried out. Evenly distributed Co nanoparticles inside the host structure enables the electrode to form thick and dominant LiF in the interphase between the ether-based electrolyte and the electrode. As a result, the Co-ZIF-C anode showed an excellent electrochemical performance with high Li-metal reversible capacity and even stable long-term cyclability with no dead Li formation (Scheme 1). To surpass the limited energy density of conventional Li-ion batteries, this approach can give more insight into the development of high energy anodes for next-generation energy storage systems.

## Data Availability

The datasets used and/or analysed during the current study are available from the corresponding author on reasonable request.

## Abbreviations

Li: Lithium
ZIF: Zeolitic imidazolate framework
Zn: Zinc
Co: Cobalt
LiF: Lithium fluoride
SEI: Solid electrolyte interphase
FESEM: Field emission scanning electron microscope
TEM: Transmission electron microscope
XRD: X-ray powder diffraction
XPS: X-ray photoelectron spectroscopy
LiTFSI: Lithium bis(trifluoromethanesulfonyl)imide
DOL: 1,3-Dioxolane
DME: Dimethyl ether
LiNO_3_: Lithium nitrate
LFP: Lithium iron phosphate
C: Carbon
N: Nitrogen
F: Fluorine
